# A cross-sectional study of echocardiographic characteristics of patients diagnosed with SARS-CoV-2 delta strain

**DOI:** 10.21542/gcsp.2023.19

**Published:** 2023-08-01

**Authors:** Amit Varshney, Ramakant Rawat

**Affiliations:** 1 Department of Emergency Medicine, Kanti Devi Medical College Hospital and Research Center, Mathura, Uttar Pradesh, India; 2 Department of Medicine, U.P. University of Medical Sciences, Safai, Etawah, Uttar Pradesh, India

## Abstract

Background: The delta variant of SARS-CoV-2 has been associated with increased mortality and multi-organ failure, affecting various systems in the body. Cardiovascular manifestations including arrhythmias, heart failure, myocarditis, myocardial damage, and thromboembolism are commonly observed in patients infected with the delta variant.

Materials and methods: This study enrolled 106 individuals who tested positive for the delta strain of SARS-CoV-2 using real-time RT-PCR between May 25, 2020, and October 15, 2021. All patients underwent 2-D echocardiography, and based on the severity of their infection, were divided into two groups: serious and non-serious.

Results: Univariate correlation analysis showed significant positive correlations between right ventricular (RV) diameter and hs-TnI and D-dimer levels. Conversely, left ventricular ejection fraction (LVEF) was negatively correlated with hs-TnI, C-reactive protein (CRP), and D-dimer levels. Additionally, RV fractional area change (RV-FAC) showed a negative correlation with D-dimer and hs-TnI levels but not with CRP levels.

Discussion: RV dysfunction has been identified as an important predictor of mortality in various patient populations, including those infected with the delta variant of SARS-CoV-2. A significant proportion of severe delta variant cases require mechanical ventilation, which can have hemodynamic effects on the ventricular performance. Mechanical ventilation can increase pulmonary arterial pressure and worsen right heart dysfunction, especially when lung-protective ventilation strategies are not optimized.

Conclusions: Our study highlights that patients with severe delta variants, particularly those with cardiac injury, may exhibit biventricular systolic dysfunction. Echocardiographic parameters such as LVEF, RV diameter, and RV-FAC were found to be associated with laboratory markers of poor prognosis, including elevated hs-TnI, CRP, and D-dimer levels. 2-D echocardiography can be a valuable tool in identifying early signs of ventricular dysfunction, aiding in the management of this patient population.

## Introduction

The delta variant, also known as the B.1.617.2 variant, is a strain of the SARS-CoV-2 virus responsible for the second wave of COVID-19 infections. It was initially identified in India on October 5, 2020, and officially named the delta variant on May 31, 2021^1^. By November 22, 2021, it was detected in over 179 countries, indicating its widespread global circulation. In June 2021, the World Health Organization (WHO) identified the delta variant as the dominant strain of SARS-CoV-2 globally^2^. The delta variant has been associated with increased mortality and multi-organ failure, affecting various systems, such as the cardiac, respiratory, renal, hepatic, neurological, and intestinal systems^3^. Cardiovascular manifestations, including different types of arrhythmias, left heart failure, myocarditis, myocardial damage, and arterial and venous thromboembolisms, are commonly observed in patients affected by the delta variant of SARS-CoV-2. Myocardial necrosis and ischemia can lead to impaired ventricular function, further increasing the risk of mortality in these patients^4^.

A valuable noninvasive test for evaluating hemodynamic status and cardiac function is 2-D echocardiography, which has become particularly relevant in SARS-CoV-2 delta variant patients with multi-organ involvement and hemodynamic instability. However, the routine use of two-dimensional echocardiography in all patients with the SARS-CoV-2 delta variant is not recommended due to the risk of infection, and comprehensive studies on two-dimensional echocardiography variables in patients with SARS-CoV-2 delta variants are currently lacking. Therefore, the aim of this study was to examine two-dimensional echocardiography variables and their potential relationship with disease severity in patients with the SARS-CoV-2 delta variant.

## Material and Methods

### Study population

A total of 106 individuals who tested positive for the SARS-CoV-2 delta strain using real-time RT-PCR were enrolled in the study between May 25, 2020, and October 15, 2021. Two-dimensional echocardiography was performed for all patients. Patients were divided into two groups based on the severity of their infection: serious and non-serious.

#### Exclusion criteria:

• <18 years of age.
• History of cardiac disease (i.e., heart failure, valvular heart, or coronary artery disease).
• History of chronic obstructive pulmonary disease, atrial fibrillation, or
• end-stage hepatic or renal failure.

#### Data collection:

Data collection was conducted retrospectively, and comprehensive details regarding patient demographics, laboratory investigations, and clinical features were obtained from clinical data. High-resolution computed tomography (HRCT) images of the lungs were acquired using a communication system and picture archiving. Furthermore, laboratory outcomes upon admission, including complete blood count, comprehensive biochemistry, including C-reactive protein (CRP), High-Sensitivity Troponin I (hs-TnI), serum electrolytes, liver and kidney function tests, and D-dimer, were meticulously documented.

#### Transthoracic 2-D echocardiography:

The 2-D echocardiography examinations were carried out in our department using an Echo transducer ( Vivid™ E95; GE Healthcare) to estimate apical and parasternal images, including M-mode, 2D, and Doppler echocardiography, by a patient lying in the left lateral decubitus position. Following the methods recommended by the American Society of Echocardiography (ASE), echocardiographic pictures were taken using the four standard views, namely apical (2- and 4-chamber), as well as parasternal (short- and long-axis).

• LVEDD (Left Ventricular End-Diastolic Diameter): Normal range varies based on age and body size, but typically around 35–45 mm.
• LVESD (Left Ventricular End-Systolic Diameter): Normal range varies based on age and body size, but is typically around 25–35 mm.
• LVEF (Left Ventricular Ejection Fraction): Normal range is typically >55–60%.
• Left atrial diameter during end-systole: Normal range varies, but typically <40 mm.
• Right ventricular dilatation (based on the left atrial diameter at the tricuspid annulus): A reading of more than 42 at the base may indicate right ventricular dilatation.
• RV mid-dimension of more than 35 indicates right ventricular dilatation.
• Right atrial enlargement: Long axis RA dimension >40 mm may indicate right atrial enlargement.
• TAPSE (Tricuspid Annulus Plane Systolic Excursion): Normal range is typically >16–18 mm.
• RV-FAC (Right Ventricular Fractional Area Change): Normal range is typically >38–45%.
• TDI-Derived Tricuspid Lateral Annular Systolic Velocity(S’): Abnormal cutoff <9.5 cm/s.

### Definitions

The severe SARS-CoV-2 delta strain group was defined based on the presence of any of the following criteria.

1. Respiratory distress, categorized by a respiratory rate of ≥ 30 breaths per minute;
2. resting SpO2 (oxygen saturation) ≤ 93%;
3. PaO2/FiO2 ratio (ratio of arterial oxygen partial pressure to fractional inspired oxygen) ≤ 300 mmHg; or
4. presence of serious complications such as respiratory failure requiring septic shock, mechanical ventilation (MV), and/or MODS (multiple organ dysfunction syndrome) necessitating ICU admission.

Elevated hs-TnI levels higher than the 99th percentile upper reference limit were used as the criteria for defining cardiac injury.

### Statistical analysis

Statistical analysis was performed using SPSS version 21.0 for Windows (SPSS Inc., Chicago, IL, USA). The Kolmogorov–Smirnov test was used to assess the null hypothesis of a normal distribution for each dataset. Continuous data are reported as the mean ± standard deviation. Percentages were used to express statistical data. The Chi-square test was used to analyze variations in categorical variables between groups. Unpaired samples were compared using various tests such as the Mann–Whitney U test or Student’s *t*-test. Spearman’s correlation analysis was conducted to assess the relationships among variables, considering the normality of the data. Multiple linear regression analyses were performed using a stepwise technique to identify independent variables that significantly affected the dependent variable. Multicollinearity among the independent variables was assessed using the Variance Inflation Factor (VIF), with a VIF value exceeding 3.0, which was considered indicative of collinearity. The confidence level for this study was set at 95%. Statistical significance was determined using a 2-sided test with a *p*-value <0.05.

**Table 1:** Demographic and clinical characteristics of serious and non-serious patient.

	Serious (*n* = 54)	Non-serious (*n* = 52)	*p*-value
Age (years)	60.3 ± 11.7	59.4 ± 17.4	0.001
Male, n (%)	29 (53%)	24 (46%)	0.484
BMI (kg/m^2^)	30.1 ± 5.6	28.1 ± 5.3	0.312
Fever, n (%)	28 (52%)	25 (48%)	0.609
Cough, n (%)	36 (67%)	32 (61%)	0.599
Shortness of breath, n (%)	33 (61%)	11 (21%)	<0.001
HT, n (%)	28 (52%)	10 (18%)	0.001
DM, n (%)	13 (24%)	5 (9%)	0.082
HLD, n (%)	8 (14%)	4 (7%)	0.212
Smoking, n (%)	34 (63%)	33 (62%)	0.954
HR, beats/min	88.1 ± 15.7	78.7 ± 13.2	0.076
SBP (mmHg)	124.5 ± 12.6	118.3 ± 9.7	0.089
DBP (mmHg)	81.4 ± 4.6	76.5 ± 9.2	0.413
*Laboratory findings on admission*			
Hemoglobin (g/L)	10.9 ± 2.8	11.5 ± 2.1	<0.001
WBC (10^3^/ μL)	6.9 (5.1–11.9)	5.9 (4.1–7.2)	0.004
Creatinine (mg/dL)	0.9 (0.6–1.2)	0.9 (0.5–1.1)	0.498
Sodium (mmol/L)	138.1 ± 5.9	136.2 ± 3.1	0.069
Potassium (mmol/L)	4.2 ± 0.7	4.3 ± 0.5	0.214
Glucose (mg/dL)	152.6 ± 61.2	119.2 ± 39.5	0.007
CRP (mg/dL)	108 (41–179)	22 (12–81)	<0.001
Hs-TnI (pg/mL)	22 (7–91)	8 (2–15)	0.005
D-dimer (ng/mL)	1205 (350–2650)	301 (31–504)	<0.001
CK-MB (ng/mL)	2.5 (1.4–3.9)	1.1 (0.8–2.2)	0.079
O_2_ saturation, %	84.7 ± 4.9	94.5 ± 2.1	<0.001
*Clinical parameters*			
ICU, n (%)	37 (68%)	–	–
MV, n (%)	32 (60%)	–	–
Non-Invasive Mechanical Ventilation (CPAP, BIPAP, AVAPs, etc.)	52(96%)	7(13%)	<0.001
Hospital stay (days)	13.1 ± 5.1	7.6 ± 5.1	<0.001

**Notes.**

BMI Body mass index HT hypertension DM diabetes mellitus HLD hyperlipidemia HR heart rate SBP systolic blood pressure DBP diastolic blood pressure WBC white blood cell CRP C-reactive protein hs-TnI high sensitive troponin I CK creatinine kinase ICU intensive care unit MV mechanical ventilation

**Table 2:** Comparison of 2-D transthoracic echocardiographic parameters in the study population.

	Serious (*n* = 54)	Non-serious (*n* = 52)	*p*-value
***Left heart findings***			
Systolic functions			
LVEF (%)	51.6 ± 8.8	59.9 ± 5.1	<0.001
LVEDD (mm)	46.2 ± 4.9	43.6 ± 3.4	0.031
LVESD (mm)	32.9 ± 6.1	29.2 ± 3.9	0.001
LV mass, g/m^2^	93.7 ± 5.9	91.4 ± 3.9	0.069
LA (mm)	41.2 ± 4.8	32.7 ± 4.2	<0.001
Diastolic functions			
E (cm/s)	58.9 ± 11.7	87.9 ± 21.9	<0.001
A (cm/s)	71.5 ± 12.2	66.5 ± 15.4	0.128
E/A ratio	0.8 ± 0.4	1.3 ± 0.5	<0.001
e’ Septal, cm/s	5.8 ± 2	7.2 ± 2	0.10
e’ Lateral, cm/s	6.9 ± 2	9.1 ± 3	0.28
E/e’ average	8.3 ± 4	11.5 ± 8	0.71
***Right heart findings***			
RV (mm)	36.9 ± 5.6	31.5 ± 6.2	0.002
RV ≥ 42 mm, n (%)	13 (24%)	3 (5%)	0.006
RV mid dimension ≥ 35 mm, n (%)	9(16%)	1(2%)	0.004
RA (mm)	39.9 ± 7.3	36.8 ± 6.6	0.023
TAPSE (mm)	20.1 ± 4.3	21.4 ± 3.6	0.126
TAPSE ≤ 16 mm, n (%)	11 (25%)	4 (8%)	0.016
RV-FAC, %	40.1 ± 3.9	43.8 ± 7.3	<0.001
TDI S’, cm/s	12.8 ± 3.5	14.2 ± 2.9	0.324
PA, mm	20.7 ± 2.7	22.2 ± 2.9	0.413
sPAP, mmHg	34.2 ± 7.8	29.5 ± 8.1	0.039
sPAP ≥ 35 mmHg, n (%)	21 (39%)	8 (15%)	0.013
IVC (mm)	15.3 ± 4.5	11.9 ± 3.0	<0.001
Pericardial effusion, n (%)	14 (26%)	0 (0%)	–

**Notes.**

LVEF left ventricular ejection fraction LVEDD left ventricular end diastolic diameter LVESD left ventricular end systolic diameter LA left atrial RV right ventricular RA right atrial TAPSE tricuspid annular plane systolic excursion RV-FAC right ventricular fractional area change TDI S’ tissue Doppler imaging systolic wave S’ velocity PA pulmonary artery sPAP systolic pulmonary artery pressure IVC inferior vena cava

**Table 3:** Correlation of echocardiographic findings with prognostic laboratory parameters.

	hs-TnI	D-dimer	CRP
RV diameter			
Correlation coefficient	**0.703** Moderate positive collinearity	**0.606** Moderate positive collinearity	**0.172** Weak positive collinearity
*p-value*	<0.001	<0.001	0.108
LVEF			
Correlation coefficient	** **−**0.332** Moderate negative collinearity	** **−**0.429** Moderate negative collinearity	** **−**0.299** Weak negative collinearity
*p-value*	<0.001	<0.001	0.002
RV-FAC			
Correlation coefficient	** **−**0.614** Moderate negative collinearity	** **−**0.655** Moderate negative collinearity	** **−**0.105** Weak negative collinearity
*p-value*	<0.001	<0.001	0.376

**Notes.**

RV right ventricular LVEF left ventricular ejection fraction RV-FAC right ventricular fractional area change hs-TnI high-sensitive troponin I CRP C-reactive protein

## Results

Table 1 displays the anthropometric and clinico-pathological characteristics of the 106 patients included in this study. Among the total participants, 52 patients were classified as having a serious condition based on predefined criteria, and they were older than the 54 patients in the non-serious group. No statistically significant differences were found between the two groups in terms of body mass index (BMI), sex, smoking, diabetes mellitus (DM), and hyperlipidemia. However, hypertension was more prevalent in the severe group. Furthermore, no statistically significant differences were observed in the heart rate, blood pressure (both systolic and diastolic), serum electrolyte levels, creatinine levels, and CK-MB (Creatine-Kinase MB) levels between the two groups. Conversely, significant differences were noted in hemoglobin levels, total leukocyte counts (TLC), blood glucose levels, CRP levels, hs-TnI levels, and D-dimer levels between the two groups. Findings consistent with typical COVID pneumonia on HRCT lung images were more frequently observed in the severe group (92% vs. 74%). Additionally, oxygen saturation levels were lower in this group of patients. The use of noninvasive ventilation (CPAP, BIPAP, AVAPs, etc.) was significantly higher in the serious group (96%) compared to the non-serious group (13%).

**Figure 1.**
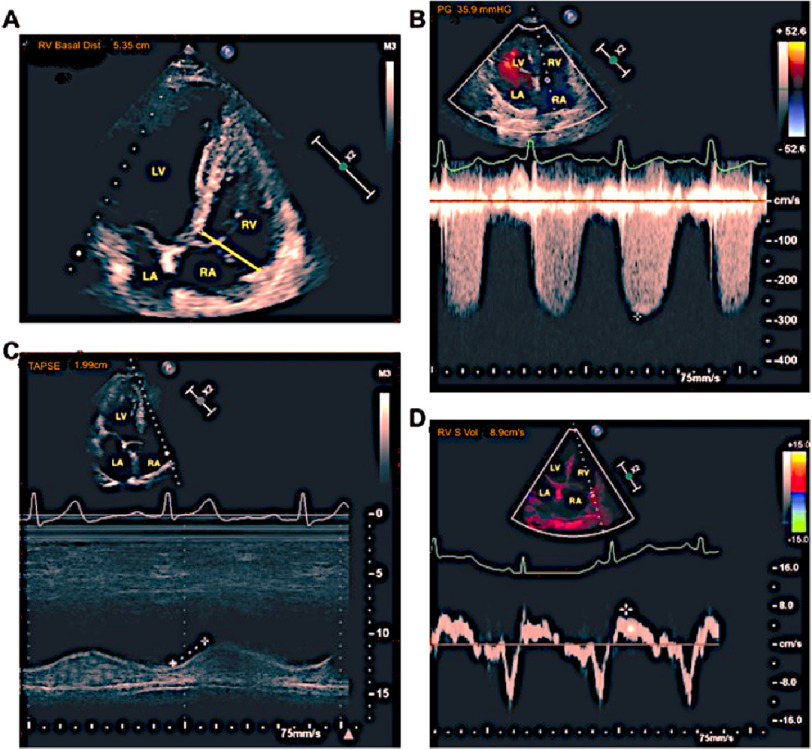
Two-dimensional transthoracic echocardiography of a patient with right ventricular dysfunction with serious SARS-CoV-2 delta strain patients. (A) Enlarged right ventricle (RV). (B) Increased systolic pulmonary artery pressure (sPAP). (C) Reduced tricuspid annulus plane systolic excursion (TAPSE). (D) Decreased TDI-derived tricuspid lateral annular systolic velocity (*S*′).

Table 2 presents a comparison of the findings from two-dimensional echocardiography between the serious and non-serious groups. In the serious patient group, diastolic dysfunction, known to occur prior to systolic dysfunction in the cascade of myocardial injury, was evident, as indicated by lower E wave velocity, LVEF, RV-FAC, and E/A ratio (Figure 1). Conversely, higher values were observed for LVEDD, LVESD, and left atrial diameter in the serious group. Additionally, the diameters of the right atrium (RA) and inferior vena cava (IVC) and systolic pulmonary artery pressure (sPAP) were higher in the serious group than in the non-serious group. The RV mid-dimension, which is a valuable variable in ventilated patients and those with COVID infections, was also higher in the serious group. It is worth noting that tricuspid annular plane systolic excursion (TAPSE) values were almost identical between the two groups. Furthermore, pericardial effusion was more frequently observed in the serious group (26% vs. 0%), indicating a significant difference between the two groups.

Univariate correlation analysis (Table 3) examined the association between right ventricular (RV) diameter, LVEF, and RV-FAC with D-dimer, hs-TnI, and CRP levels. The results revealed significant positive correlations between RV diameter and hs-TnI levels, as well as D-dimer levels. In contrast, LVEF was negatively correlated with hs-TnI, CRP, and D-dimer levels. Furthermore, RV-FAC demonstrated a negative correlation with D-dimer and hs-TnI levels, which was statistically significant, but not with CRP level.

## Discussion

The SARS-CoV-2 delta strain is known to cause multi-organ dysfunction, and the presence of cardiac involvement is associated with poorer outcomes. Although two-dimensional echocardiography is a valuable tool for evaluating cardiac function and hemodynamic status in these patients, it is not frequently used because of the risk of disease transmission. Nonetheless, two-dimensional echocardiography can provide important noninvasive data on disease severity. Furthermore, it can be utilized to rule out obstructive issues such as pericardial effusion resulting in cardiac tamponade and pulmonary thromboembolism, as well as hypotension due to hypovolemic shock (indicated by collapsed IVC and low cardiac output). Recent studies have highlighted the significance of two-dimensional echocardiography in patients with sepsis requiring mechanical ventilation^5–6^. In the serious group, there was evidence of diastolic dysfunction, which is known to occur before systolic dysfunction, in the progression of myocardial injury. This was reflected by the lower values of E wave velocity, LVEF, RV-FAC, and E/A ratio. These findings regarding diastolic dysfunction have implications for morbidity, mortality, and poor patient outcomes. These studies have emphasized that approximately one-third to half of sepsis patients develop reduced LVEF. In the early stages of sepsis, two-dimensional echocardiography shows hyperdynamic cardiac function, characterized by increased cardiac output and LVEF due to systemic inflammatory responses. However, in the late stages, severe hypoxia and inflammation can lead to extensive myocardial depression.

Injury to the myocardium has been associated with a worse prognosis in patients with the SARS-CoV-2 delta strain. Findings such as regional wall motion abnormalities or global hypokinesia of the left ventricle, suggested to be caused by inflammation and hypoxic myocardial injury, have been observed, which can lead to respiratory distress. Contemporary studies emphasize the importance of a reduction in RV longitudinal strain (<23%), which can be associated with a poor prognosis, even in patients with normal LVEF^7–9^. In serious patient groups with deteriorating clinical conditions and elevated markers of myocardial injury (such as high-sensitivity cardiac troponin I), along with an increased RV mid-dimension, which is a significant parameter for patients on mechanical ventilation and those with COVID infections, poorer RV function was observed compared to patients without myocardial injury or those in the non-serious group.

This study differs from previous studies in that we observed low LVEF and left ventricle (LV) dilatation, along with a decrease in RV-FAC, in serious patients with myocardial injury^10–12^. In contrast to other studies that reported normal or increased LVEF with dilated and reduced RV function, our findings showed reduced LVEF and RV-FAC, as well as increased LV and RV dimensions, and a higher frequency of LV diastolic dysfunction in serious patients, including those who required mechanical ventilation. However, within the serious group, echocardiographic indices were similar regardless of the mode of ventilation used, although RV function and LV systolic function were still poorer than those in the non-serious group.

RV dysfunction has been identified as a significant predictor of mortality in various patient populations, including those infected with the SARS-CoV-2 delta strain^13–15^. The utilization of noninvasive ventilation (CPAP, BIPAP, AVAPs, etc.) was significantly higher in the serious group (96%) compared to the non-serious group (13%). This approach was based on our previous experiences, as we attempt noninvasive ventilation in every patient before resorting to mechanical ventilation.

A considerable proportion of serious SARS-CoV-2 delta strain cases require mechanical ventilation, and it is well-established that mechanical ventilation can have significant hemodynamic effects on ventricular performance. Mechanical ventilation can elevate pulmonary artery pressure and worsen right heart dysfunction, especially when lung-protective ventilation strategies are not optimally implemented. Therefore, it is recommended that the initiation of mechanical ventilation be delayed for as long as possible in every patient to minimize the potential adverse effects of mechanical ventilation on ventricular function.

## Conclusion

In our study, we observed that patients with severe SARS-CoV-2 delta strain, particularly those with cardiac injury, may exhibit biventricular systolic dysfunction. Echocardiographic variables such as LVEF, RV mid-dimension, and RV-FAC were found to be related to laboratory markers of poor prognosis, including elevated hs-TnI, CRP, and D-dimer levels. 2-D echocardiography can be valuable in identifying early indications of ventricular dysfunction, which can aid in guiding therapy in this patient population. Additionally, 2-dimensional echocardiography can be valuable in identifying global or regional wall motion abnormalities of the LV in particular patients and in exhibiting acute RV dysfunction in the presence of acute respiratory distress syndrome (ARDS), particularly in the presence of hemodynamic instability.

## Limitations

Like any study, our research had several limitations. One significant limitation was the absence of a control group, which may have affected the study design. Another drawback of our research was that the information was acquired from a single center, and the sample size was comparatively small; therefore, the generalizability of the findings may be limited. Additionally, measurements of important hemodynamic parameters (such as cardiac index, longitudinal strains, and cardiac output) for both the left & right ventricles were not available, which could have provided more comprehensive insights into ventricular function. Moreover, the lack of pre-disease and follow-up two-dimensional echocardiography data for patients related to echocardiography parameters was also a limitation of the study, further reducing the ability to draw robust conclusions. Future studies with larger sample sizes, multicenter designs, and comprehensive hemodynamic measurements are warranted to further elucidate the cardiac manifestations of the SARS-CoV-2 delta strain.
